# A tissue-level phenome-wide network map of colocalized genes and phenotypes in the UK Biobank

**DOI:** 10.1038/s42003-022-03820-z

**Published:** 2022-08-20

**Authors:** Ghislain Rocheleau, Iain S. Forrest, Áine Duffy, Shantanu Bafna, Amanda Dobbyn, Marie Verbanck, Hong-Hee Won, Daniel M. Jordan, Ron Do

**Affiliations:** 1 The Charles Bronfman Institute for Personalized Medicine, Icahn School of Medicine at Mount Sinai, 1468 Madison Avenue, New York, NY 10029 USA; 2 Department of Genetics and Genomic Sciences, Icahn School of Medicine at Mount Sinai, 1425 Madison Avenue, New York, NY 10029 USA; 3 Medical Scientist Training Program, Icahn School of Medicine at Mount Sinai, 1468 Madison Avenue, New York, NY 10029 USA; 4 Pamela Sklar Division of Psychiatric Genomics, Icahn School of Medicine at Mount Sinai, 1470 Madison Avenue, New York, NY 10029 USA; 5 UR 7537 – BioSTM, Biostatistique, Traitement et Modélisation des données biologiques, Faculté de Pharmacie de Paris, Université de Paris, 4 avenue de l’Observatoire, 75270 Paris, France; 6 Samsung Advanced Institute for Health Sciences and Technology (SAIHST), Sungkyunkwan University, Samsung Medical Center, Seoul, South Korea

**Keywords:** Medical genomics, Gene expression

## Abstract

Phenome-wide association studies identified numerous loci associated with traits and diseases. To help interpret these associations, we constructed a phenome-wide network map of colocalized genes and phenotypes. We generated colocalized signals using the Genotype-Tissue Expression data and genome-wide association results in UK Biobank. We identified 9151 colocalized genes for 1411 phenotypes across 48 tissues. Then, we constructed bipartite networks using the colocalized signals in each tissue, and showed that the majority of links were observed in a single tissue. We applied the biLouvain clustering algorithm in each tissue-specific network to identify co-clusters of genes and phenotypes. We observed significant enrichments of these co-clusters with known biological and functional gene classes. Overall, the phenome-wide map provides links between genes, phenotypes and tissues, and can yield biological and clinical discoveries.

## Abstract (web summary)

A phenome-wide genomic analysis on the UK Biobank cohort links >9,000 co-localized genes and >1,400 human phenotypes across a broad selection of human tissues, representing a useful resource for the investigation of relationships between gene expression, tissues, and human diseases.

## Introduction

Electronic health records (EHR)-linked biobanks coupled with genome-wide genotyping and sequencing data allows for the study of the impact of genetic variation on thousands of medical phenotypes simultaneously. Phenome-wide association analyses have been conducted in several EHR-linked biobanks and genome-wide association study (GWAS) summary statistics have been made publicly available for large biobanks such as the UK Biobank and FinnGen study. For example, GWAS summary statistics from a phenome-wide scan of the UK Biobank (UKBB)—a prospective cohort with deep genetic and rich phenotypic data collected on approximately 500,000 middle-aged individuals (aged between 40 and 69 years old) recruited from across the United Kingdom^1^—now exists and is a rich resource in the human genetics community.

Large-scale EHR-linked biobanks with available genetic data, such as the UKBB, permit the study of the relationship of tens of thousands of genes and phenotypes simultaneously. However, a major challenge is an interpretation due in large part to the complexity and heterogeneity of this wealth of data. Furthermore, there is a general lack of statistical methods available for such high-throughput analysis. Hence, only a few efforts have systematically characterized disease relationships in EHR data^2,3^.

In this study, we sought to enhance our understanding of the complex relationship of genes and phenotypes in the medical phenome by constructing a tissue-level phenome-wide network map of colocalized genes and phenotypes. Our main motivation was to create a tool for researchers to evaluate shared links between colocalized genes and a wide array of phenotypes. The approach is an extension of the PheWAS approach but instead of simply uncovering cross-phenotype associations^4^, the approach directly generates links between specific colocalized genes and phenotypes in specific tissues and identifies clusters within these shared links that reflect meaningful common causal mechanisms and/or pleiotropic genetic effects. To construct the phenome-wide map, we first generated tens of thousands of colocalized expression quantitative trait loci (eQTL) from 48 tissues of the Genotype-Tissue Expression (GTEx) v7 project^5–7^, and from ~3800 GWAS of biological and medical phenotypes from the UKBB. We then applied a bipartite (or two-mode) network approach^8,9^ followed by the biLouvain clustering method^10^, to identify networks of genes and phenotypes that co-cluster together in different tissues, giving us broad insight into the biological structure of genes, phenotypes, and tissues. Finally, we demonstrate the functionality of the phenome-wide map by highlighting co-clusters that are biologically relevant.

## Results

**Fig. 1:**
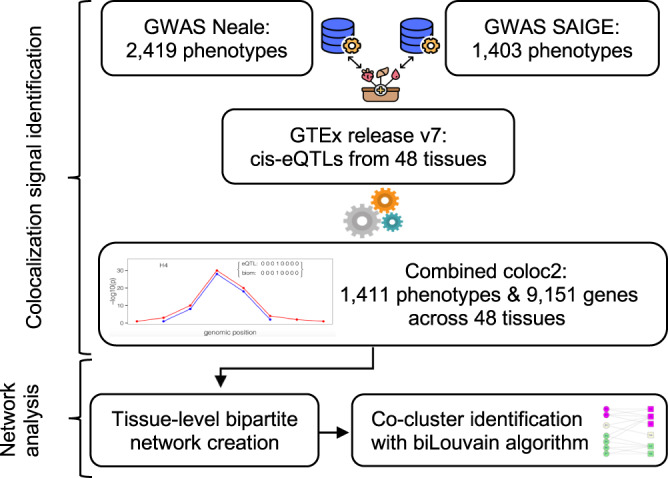
Flowchart of the study. The flowchart illustrates all the different steps of our study. GWAS genome-wide association study, GTEx Genotype-Tissue Expression, eQTL expression quantitative trait locus.

We performed three steps to generate the phenome-wide network map of genes and phenotypes: (1) identification of colocalization signals of eQTLs and GWAS loci for various continuous and binary phenotypes in 48 tissues from the GTEx project v7; (2) construction of a bipartite network using the colocalization signals to establish links between genes and phenotypes in each tissue; and (3) identification of co-clusters of colocalized genes and phenotypes in each bipartite network using the biLouvain clustering algorithm. A graphical flowchart of the study is shown in Fig. 1.

### Identification of colocalization signals of eQTL and GWAS loci in multiple tissues

We used coloc2^11^, along with GWAS summary association statistics available for 3822 phenotypes in UKBB and eQTL data to identify colocalization signals in 48 tissues from the GTEx project. Before running coloc2, we performed stringent quality control (QC) on the phenotypes in UKBB. We removed phenotypes related to cause of death and case–control phenotypes with less than 1250 cases (or controls), except phenotypes showing prior gene/locus association as reported in the NHGRI-EBI GWAS catalog. In Neale GWAS data, we retained coloc2 results for 496 continuous and binary phenotypes. In SAIGE data, we excluded case–control phenotypes with less than 200 cases, retaining coloc2 results for 915 case–control (PheCode) phenotypes. We selected variants with minor allele frequency (MAF) > 0.1% in both Neale and SAIGE datasets; in GTEX, we included cis-eQTL variants with MAF >1% from Analysis V7. We restricted our study to the list of 48 tissues (from 620 donors) having a sample size of at least 80. In total, after QC (see “Methods”), we identified 9151 unique colocalized genes for 1411 unique phenotypes across the 48 selected tissues. Colocalization results for each tissue are reported in Supplementary Data 1. Unsurprisingly, the number of colocalized genes and phenotypes increases with respect to the GTEx tissue sample size (from *n* = 80 for brain–substantia nigra to *n* = 491 for muscle − skeletal), reflecting the enhanced statistical power of the method to uncover colocalized genes (see Supplementary Fig. 1).

### Construction of tissue-level bipartite networks

Using the colocalized data of 9151 genes and 1411 phenotypes, we next created a bipartite network for each tissue. In brief, a bipartite network—also called a two-mode network—is a network in which nodes of one mode (i.e., type) are only connected to nodes of the other mode, as opposed to a unipartite (or one-mode) network commonly found in the network literature. In our colocalization results, phenotypes are not directly connected to other phenotypes, but could only be indirectly connected to each other through genes they share, while genes are indirectly connected to other genes if they appear in the same phenotype. In Supplementary Fig. 2a, a typical graphical representation of a bipartite network is displayed, comprised of seven phenotypes and six genes. Associations between genes and phenotypes are indicated by links (or edges) between them.

For each tissue, Supplementary Data 2 displays the number of unique colocalized genes and phenotypes, along with the number of links between the two sets. When all 48 tissues are aggregated, there are 9151 unique colocalized genes and 1411 unique phenotypes, with 25,710 unique links between the two sets. We aggregated the tissues using an unweighted approach which means that if a link between a gene and a phenotype was found in more than one tissue, we counted this link only once. In fact, we observed that the majority of links between a given gene and a given phenotype are observed in one single tissue, but a few links are present in all 48 tissues (see Supplementary Fig. 3 and Supplementary Data 3).

To characterize and compare colocalization results across tissues, we computed the average degree of colocalized genes and phenotypes in each tissue. The degree of a given gene (respectively, phenotype) is simply the number of unique phenotypes (respectively, genes) connected to it in the network, the average being taken over the total number of genes (respectively, phenotypes)^12^. The average degree for both genes and phenotypes does not vary much across tissues (see Supplementary Data 2), although it increases with larger tissue sample size. When aggregating all 48 tissues, each gene is connected to an average of ~2.8 phenotypes, while each phenotype is connected to an average of ~18.2 genes. The fact that the majority of gene and phenotype links are observed in a single tissue and not across all tissues, but the average degree of genes and phenotypes does not vary across tissues, suggests an architecture where tissue-specific gene regulatory mechanisms drive GWAS loci, and the size and structure of these mechanisms are largely similar across different tissues.

### Identification of co-clusters in tissue-level bipartite networks

To identify structure within the phenome-wide map, we applied a clustering algorithm, called biLouvain, which extends the well-known unipartite Louvain clustering algorithm^10^. This algorithm efficiently identifies co-clusters of non-overlapping genes and phenotypes by maximizing a bipartite modularity measure (see “Methods” for details). Supplementary Fig. 2b illustrates co-clusters identified by the biLouvain algorithm in the bipartite network of Supplementary Fig. 2a.

**Fig. 2:**
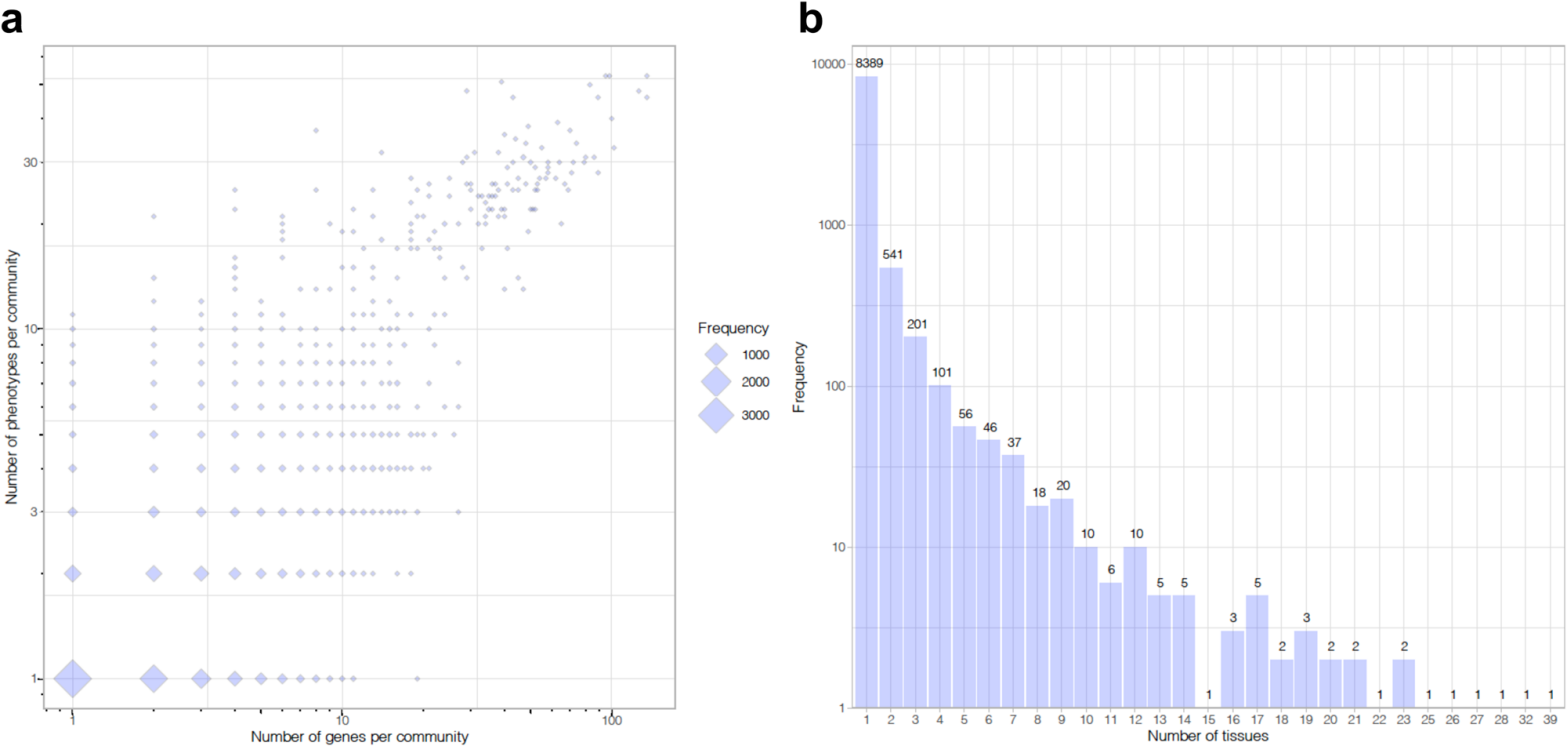
Characteristics of biLouvain co-clusters across tissues. **a** Number of genes and phenotypes per co-cluster identified by the biLouvain algorithm. Diamonds are proportional to the frequency of co-cluster size across all 48 tissues. Both axes are displayed on the log scale. **b** Number of unique co-clusters and how many times they appear in a given number of tissues. *y* axis is displayed in log scale.

We applied the biLouvain algorithm to identify co-clusters in each of the tissue-level bipartite networks. We identified a large number of co-clusters, ranging from 218 co-clusters for tissue brain-anterior cingulate cortex (BA24) to 314 co-clusters for adipose–subcutaneous. Across all bipartite networks, we observed that the majority of co-clusters had a small number of genes and phenotypes, on one hand, whereas a few co-clusters had a large number of genes and phenotypes, on the other hand (Fig. 2a, Supplementary Fig. 4 and Supplementary Data 4). Across the 48 tissues, the vast majority of co-clusters (8389/9472 = 88.6%) were found in only one tissue (Fig. 2b). Hence, the structure of the phenome-wide map involves hundreds of isolated tissue-specific subnetworks comprised of a small number of interrelated genes and phenotypes. Large co-clusters were also identified, although these are the exception rather than the norm. The complete list of genes and phenotypes per co-cluster in each tissue is provided in Supplementary Data 4.

### Enrichment analysis of co-clusters with biological and functional gene classes

**Fig. 3:**
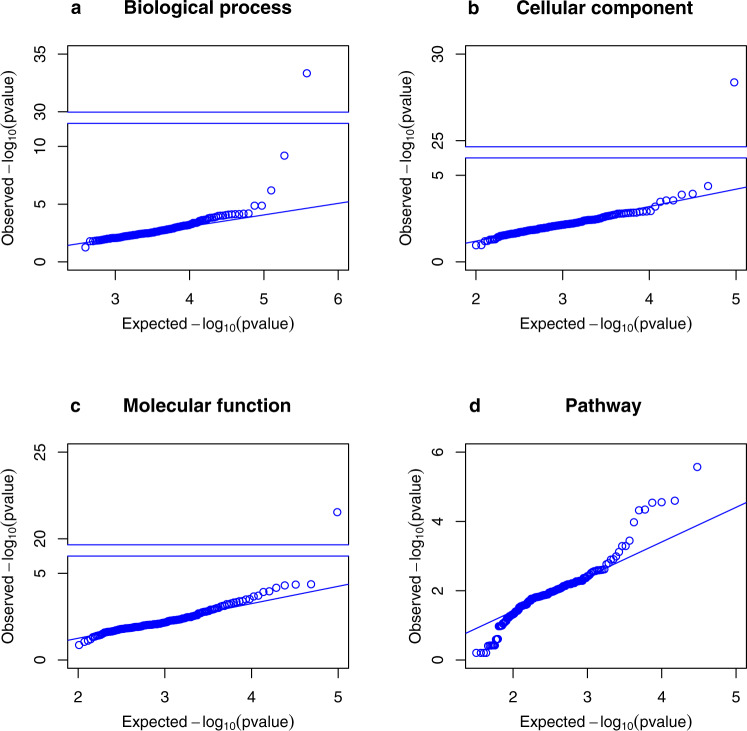
PANTHER enrichment analysis of selected biLouvain co-clusters across tissues. Each panel represents a PANTHER annotation type: (**a**) Biological process; (**b**) Cellular component; (**c**) Molecular function; and (**d**) Pathway. In each panel, the observed minimal *P* value across all GO terms is plotted against the expected minimal *P* value under the null hypothesis of no enrichment for all 183 co-clusters selected. Some plots (**a**–**c**) show a breakdown in the *y* axis to help display very small *P* values.

To demonstrate the functionality of the phenome-wide map, we tested if the identified biLouvain co-clusters were enriched with known biological and functional gene classes. We selected 183 co-clusters consisting of 10 genes or more, and performed enrichment analysis using PANTHER^13,14^ on four different annotation types: Biological process (2064 gene ontology (GO) terms), Cellular component (520 GO terms), Molecular function (532 GO terms), and 164 different Pathways. For each co-cluster and each annotation type, we selected the minimal *P* value of all Fisher overrepresentation tests, and plotted it against the expected minimal *P* value under the null hypothesis of no enrichment (see “Methods” for details). All four annotation types demonstrated significant enrichment (Fig. 3). We observed enrichment in GO terms related to (i) antibody-mediated immune response, upregulation response to biotic stimulus, glutathione metabolism, zymogen activation, downregulation of blood pressure, and cellular nitrogen compound metabolism in seven co-clusters in the Biological process annotation; (ii) outer surface of cytoplasmic membrane, and obsolete intracellular part in two co-clusters in the Cellular component annotation; (iii) signaling receptor binding, metallopeptidase activity, zinc ion binding, NADH-dependent glyoxylate reductase, and phosphatase activity in five co-clusters in the Molecular function annotation; and (iv) toll-like receptor signaling pathway, muscarinic acetylcholine receptor 2 and 4 signaling pathway, serine and glycine biosynthesis, and heterotrimeric G-protein signaling pathway-Gq alpha and Go alpha mediated pathway in six co-clusters in the Pathway annotation.

As an example, the most significant pathway detected by PANTHER is a toll-like receptor signaling pathway for co-cluster 111 comprised of hypothyroidism/myxedema and levothyroxine sodium medication, and genes *TLR1*, *TLR6,* and *TLR10* in the cells—EBV-transformed lymphocytes tissue (Fig. 3a, Table 1, and Supplementary Fig. 5). Toll-like receptor 1 (*TLR1*), 6 (*TLR6*), and 10 (*TLR10*) genes are located in the same gene cluster on chromosome 4p14, and they play a fundamental role in pathogen recognition and activation of innate immunity^15^. Previous studies have shown that *TLR1* and *TLR10* are linked to Graves’ disease^16^ and Hashimoto’s disease^17^, which are clinical subtypes of autoimmune thyroid diseases. Furthermore, variants in *CD226*^18^, and *RASGRP1*^19^ were found to be associated with autoimmune thyroid diseases and with thyroid preparations (H03A medication class, which comprises levothyroxine sodium)^20^.

**Table 1:** Selection of interesting co-clusters in relevant tissues.

Co-cluster/tissue	Nb of phenos	Nb of genes	Phenotype^‡^	Gene	Biological function summary
Co-cluster 111^†^ /cells—EBV - transformed lymphocytes	4	13	Hypothyroidism/myxedema | Med: levothyroxine sodium	*RP11-102L12.2*, *CD226*, *SYNGR1, FAM98B, RASGRP1, FAM114A1, MDGA1, TLR6, RRP15, PRKXP1, RP11-526I2.5, TLR10, TLR1*	*CD226*, *RASGRP1*, *TLR1, TLR6, TLR10*: immune function and response, including lymphocyte activity; associated with autoimmune thyroid disease.
Co-cluster 161 /heart-left ventricle	6	12	Angina pectoris | Ischemic heart disease (chronic, other) | Coronary atherosclerosis | Acute pain	*VPS11, MRPL9, GGCX, RP11-114H24.5, RP11-110I1.14, HMBS, DHX36, STAG1, ZNF334, ZNF663P, MKRN7P, EHBP1L1*	*GGCX*: activates coagulation factors. *DHX36*, *STAG1*: cardiac development. Abnormal clotting and cardiac defect are associated with ischemic heart disease.
Co-cluster 27 /adipose– subcutaneous	11	18	High cholesterol | Hayfever/allergic rhinitis | Breast cancer | Diabetes (Type 1, Type 2) | Diabetic retinopathy | Illness mother: diabetes | Med: metformin	*JAZF1, ANK1, WFS1, GINS4, IRS1, FARSA, CALR, RP11-395N3.1, RP11-395N3.2, PRRT1, RP11-686O6.2, AC010883.5, ZNF703, RP11-419C23.1, ITGB6, EYA1, RP11-863K10.7, RP11-379H18.1*	*JAZF1*: lipid and glucose metabolism. *WFS1*: Wolfram syndrome (monogenic diabetes). *IRS1*: insulin response. *ZNF703*: oncogene in mammary epithelial proliferation.
Co-cluster 128 /thyroid	4	12	Nontoxic goiter (uninodular, multinodular, other)	*FOXA2, RP4-788L20.3, LINC00261, PRDM11, TGFB2, CPE, SYT13, MINOS1, LINC00887, RP11-224O19.2, CTD-2560E9.3, RP11-555G19.1*	*FOXA2*: regulates type 1 iodothyronine deodinase in thyroid hormone homeostasis *CPE*: synthesizes thyrotropin-releasing hormone; deficiency and hypothyroidism cause goiter formation.
Co-cluster 39 /artery-tibial	15	10	Angina (unstable) | Myocardial infarction | Ischemic heart disease (chronic) | Illness father: heart disease | Migraine/Headache | Med: ibuprofen, paracetamol	*PHACTR1, RP1-257A7.5, RP11-378J18.8, LRP1, C7orf10, UFL1, GJA1, EHBP1, C12orf4, MEF2D*	*PHACTR1*: vascular endothelial maintenance. *LRP1*: macrophage/vascular lipid homeostasis. *UFL1*: immune and ER-stress response. *GJA1*: cardiac gap junctions, arrhythmia, malformation. *EHBP1*: cardiomyocyte protein trafficking and excitability. *MEF2D*: cardiogenesis and cardiac remodeling. Migraine medications associated with increased risk of heart attack.
Co-cluster 43/cells - transformed fibroblasts	13	11	Asthma | Hayfever/allergic rhinitis | Eczema/dermatitis | Wheezing/whistling | Med: ventolin, seretide | Nasal polyps	*GATA3, GATA3-AS1, HHEX, RP1-102E24.8, LINC01063, SERPINB7, STAT6, ARFRP1, CRYAB, TNK2-AS1, RBM26-AS1*	*GATA3, GATA3-AS1*, *STAT6*, *CRYAB*: regulate T_h_2 cells, cytokines; associated with asthmatic and fibrotic airways. *HHEX*: fibroblast expression with increased asthma risk. *SERPINB7*: stratified squamous epithelia expression with increased allergies risk.
Co-cluster 11/whole blood	32	14	Angina (pectoris, unstable) | Ischemic HD (chronic, other) | MI | Coronary atherosclerosis | Illness father: HD, high BP | Illness mother: high BP | Father’s age at death | Hyperlipidemia/hypercholesterolemia | Med: simvastatin, atenolol, aspirin, lipitor, atorvastatin, ezetimibe	*DDAH2, FES, PSRC1, MIA3, FAM177B, OPRL1, RPS12P26, FNBP4, CELF1, GGCX, LIPA, COA6, MCL1, GRK4*	*DDAH2*: regulates nitric oxide formation; cardiac dysfunction in animal models. *OPRL1*, *PSRC1*, *MIA3*: LDL-C levels and CAD. *CELF1*, *COA6*: implicated in cardiomyopathy. *GGCX*: activates coagulation factors. *LIPA*: lipid metabolism. *MCL1*: deficiency causes atherosclerosis. *GRK4*: hypertension and cardiomyocyte injury during MI.

*Nb* number, *HD* heart disease, *MI* myocardial infarction, *BP* blood pressure, *Med* medication, *LDL-C* low-density lipoprotein cholesterol, *CAD* coronary artery disease.

^†^Co-cluster most significant in PANTHER Pathway annotation.

^‡^For the sake of simplification, we grouped phenotypes with similar names in UKBB and/or matching ICD-10 codes and PheCodes.

In addition to the PANTHER gene set enrichment analysis, we identified seven co-clusters comprised of known relationships between genes and phenotypes in relevant tissues (Table 1), providing strong biological relevance. For example, the same gene *GGCX* appears in two co-clusters in related tissues, co-cluster 161 in heart-left ventricle and co-cluster 11 in Whole Blood (Table 1 and Supplementary Figs. 6 and  7). Gamma-glutamyl carboxylase (*GGCX*) encodes an integral membrane protein of the rough endoplasmic reticulum that carboxylates glutamate residues of vitamin K-dependent proteins to gamma carboxyl glutamate. Vitamin K-dependent proteins affect a number of physiologic processes including blood coagulation, inflammation, and prevention of vascular calcification^21^. Furthermore, a meta-analysis including the UKBB data identified an intronic variant in *GGCX* associated with coronary artery disease (CAD), with inclusion or exclusion of angina^22^. Taken together, these results suggest that the identified co-clusters contain relevant biological information highlighting functional links between genes and phenotypes.

### Comparison between eQTLGen and GTEX

We compared the colocalizated loci found in GTEX with a larger gene expression level dataset. The eQTLGen Consortium identified cis-eQTLs in blood based on a meta-analysis of 37 different cohorts in up to 31,684 individuals^23^. We reran coloc2 using the same GWAS summary statistics (Neale and SAIGE) as before, but this time using cis-eQTL variants from the eQTLGen consortium (see “Methods”). All colocalization results can be found in Supplementary Data 5. As expected, more unique links between genes and phenotypes (2474) were discovered compared to links found using GTEX Whole Blood tissue (1755), most likely due to increased statistical power. However, we found 430 overlapping loci (a single colocalized locus can contain more than one gene) between GTEX and eQTLGen colocalization results in 251 distinct phenotypes (Supplementary Data 6). After running the biLouvain algorithm, we compared the clustering obtained in the colocalized loci from the eQTLGen dataset with those in the GTEX whole blood tissue. All co-clusters in the eQTLGen colocalization results are listed in Supplementary Data 7. Large co-clusters mostly contained the same phenotypes, although the colocalized genes could differ between eQTLGen and GTEX. One such large co-cluster in eQTLGen (co-cluster 42) includes many of the same phenotypes as in co-cluster 11 in GTEX Whole Blood (Table 1), and many genes are common to both co-clusters (*DDAH2, FES, PSRC1, FAM177B, RPS12P26, MCL1*).

### Colocalization with disease case sample size larger than UKBB

We assessed the effect of using GWAS summary statistics with case sample size in various diseases larger than in UKBB. Increased case sample size as observed in consortia data should in theory generate more colocalized signals than using a population-based cohort such as UKBB. To this end, we evaluated three GWAS meta-analysis case–control datasets as generated by three different consortia: coronary artery disease (CAD) from CARDIoGRAMplusC4D^24^, schizophrenia from the Psychiatric Genomics Consortium (PGC)^25^, and type 2 diabetes (T2D) from DIAGRAM^26^. We reran coloc2 by combining each of these three GWAS datasets with the same GTEX cis-eQTL dataset used before (all 48 tissues). All colocalization results are found in Supplementary Data 8 for CAD, in Supplementary Data 9 for schizophrenia, and in Supplementary Data 10 for T2D.

Regarding CAD and T2D, many colocalized loci discovered using either Neale or SAIGE datasets were replicated using the GWAS meta-analysis consortia data, even though CARDIoGRAMplusC4D^24^ defined CAD more comprehensively as either myocardial infarction, acute coronary syndrome, chronic stable angina or coronary stenosis >50% (~61,000 CAD cases) while in Neale and SAIGE, such a composite diagnosis is not available, only subphenotypes such as heart attack/myocardial infarction (Neale 20002_1075) or coronary atherosclerosis (SAIGE PheCode 411.4). In DIAGRAM^26^, the number of T2D cases (~55,000 cases, excluding the UKBB cohort) is about three times the number of cases in Neale (16,183 cases for 2443—Diabetes diagnosed by a doctor) or SAIGE (18,945 cases for PheCode 250.2—Type 2 diabetes), which enhanced the statistical power to detect more colocalized signals. By contrast, using the GWAS summary statistics dataset from the PGC^25^ largely increased the number of colocalized signals compared to using either Neale or SAIGE datasets. No colocalized signal was found with Neale (337 cases for 20002_1289—Self-reported schizophrenia) and only a few using SAIGE (571 cases for PheCode 295.1—Schizophrenia). The PGC summary statistics (which include ~37,000 schizophrenia cases, about 65 times the number of cases using SAIGE) uncovered many colocalized loci undetected by using either Neale or SAIGE: for example, genes *BNIP3L*, *CNTN4*, *THOC7*, *TRPC4*, *ZNF823*, *CLCN3*, *PAX6* were prioritized in the context of synaptic location and function from genome-wide enrichment tests in the latest PGC schizophrenia meta-analysis^27^.

## Discussion

In this study, we have constructed a tissue-level phenome-wide network map, called biPheMap, of colocalized genes and phenotypes using a bipartite network and biLouvain clustering approach on 1411 phenotypes and eQTL data from 48 tissues from the GTEx project. In the phenome-wide map, we observed the following: (1) the majority of colocalized gene and phenotype links are observed in a single tissue, implying that tissue-specific gene regulatory mechanisms drives phenotypic variation; (2) the majority of co-clusters are comprised of a small number of gene and phenotype links; (3) specific co-clusters are enriched with functional gene set annotations; (4) specific co-clusters are identified with biologically relevant gene, phenotype and tissue functions.

While most network analyses have focused on unipartite networks, this study used the less familiar bipartite approach. Many such bipartite networks have been studied in different contexts: actor-movie network in cinema industry, author-scientific paper networks in academia, pollinator-plant in ecological networks, etc., but their topological features and related metrics are unique and different from their more classical unipartite counterpart. A simpler analysis could have been proposed by projecting the bipartite network into two unipartite networks to produce a gene-gene network and a phenotype-phenotype network. However, this projection method entails a loss of information since the original links between genes and phenotypes are no longer available. Such a projection approach was employed in ref. ^28^ to create a disease-disease network where more than 500 diagnosis codes were linked on the basis of shared variant associations.

An important feature of the phenome-wide map is the exploration and discovery of co-clusters of related genes and phenotypes. So far, few community detection algorithms in bipartite networks could be run in a reasonable amount of time. One fast and precise algorithm is the biLouvain algorithm^10^ which maximizes bipartite modularity, an extension of the modularity measure found in unipartite network clustering algorithms. The biLouvain creators compared their algorithm against five state-of-the-art bipartite community detection algorithms. In their evaluation, they conclude that biLouvain is always close or equal to the maximum bipartite modularity achieved by any of the five methods while being consistently one of the fastest algorithms for the large real-world datasets tested.

Table 1 displays various examples of gene-phenotype co-clusters confirming known genetic associations and also suggesting unsuspected etiological links between phenotypes. We note that this represents only a small fraction of interesting co-clusters we chose to highlight in our paper. For example, many epidemiological and genetic studies have suggested shared loci between migraine and CAD, and one study identified gene *PHACTR1* as the strongest shared locus between the two disorders^29^. However, some co-clusters might also consist of phenotypes being observed as a consequence of another phenotype. For example, we observed lipid-lowering medications in the same co-cluster as lipid disorders.

There are numerous limitations to our study that deserve mention. First, some phenotypes are highly correlated in UKBB, and therefore, colocalized signals were sometimes redundant in our phenome-wide map. However, this redundancy provided some internal replication of the strongest colocalized signals between Neale and SAIGE association datasets, while sometimes complementing signals found in one dataset but not in the other due to different criteria of case and control definition (ICD-10 codes versus PheCodes)^30^. The PheCode scheme, as utilized in SAIGE genetic association scans, applies stringent exclusion criteria to prevent contamination by cases in the control group, which could decrease the statistical power of association tests. Second, we applied stringent quality control in our phenotype selection to avoid reporting false colocalized loci. This came at the expense of missing some loci, especially if the leading associated variant in a locus is rare (minor allele frequency <0.1%). Third, the coloc2 method assumes that at most one causal variant affects both the gene expression and the trait association at the locus under consideration. In presence of allelic heterogeneity (more than one causal variant), the false discovery rate is maintained but coloc2 might suffer a loss of power^31^. In contrast, a method such as the regulatory trait concordance (RTC) score assumes that a GWAS variant and eQTL variant located in the same genomic region delimited by recombination hotspots tag the same functional variant. In ref. ^32^, the authors proposed to compute a probability of shared functional effect, called P(shared), based on the regulatory trait concordance (RTC) score, and to compare this probability with PPH4 as generated by coloc. Interestingly, their simulation study suggests that, knowing the GWAS and eQTL *P* values for every variant in the region, coloc is a better choice as it uses all the information in the locus. Fourth, the literature cited to support the links between genes and phenotypes of co-clusters displayed in Table 1 relies heavily on genetic associations found in the GWAS summary statistics of European ancestry participants in UKBB. Incorporation of findings from diverse ancestry populations will be necessary to yield more genetic association phenotypes. Fifth, we used the bipartite network approach over the more common unipartite approach, which limited the set of tools to analyze our results. Fortunately, the bipartite network and its characteristics are gaining more attention in the network literature, and future methodological developments will expand the range of tools and analyses that could be performed in this type of networks. Sixth, the first version of biPheMap currently utilizes Neale v1 GWAS summary statistics and GTEX v7 data. Recently, more GWAS summary statistic data (e.g., Pan-UK Biobank^33^) and more recent versions of GTEX (e.g., GTEX v8^34^) have been published. Furthermore, additional large-scale phenome-wide association datasets originating from exome sequencing data (e.g., genebass^35^) have become available. We expect subsequent versions of biPheMap will incorporate these resources as they become publicly available over time. Seventh, while we recognize that the GWAS of many binary (case–control) phenotypes in UKBB are underpowered due to the reduced number of cases for many of them -hence reducing the power to detect colocalization loci- our study was more focused at identifying the strongest shared links between genes and phenotypes in a single population-based cohort. Nonetheless, to increase the number of colocalization loci, an alternative route is to include GWAS summary statistics from other consortia (which usually include more cases than UKBB), in addition to a larger gene expression level dataset, such as the whole blood eQTLGen consortium^23^. Our colocalization analyses using larger case–control GWAS and/or eQTL datasets independently confirmed many colocalized loci found in the biPheMap while uncovering additional ones that were missed due to the lower number of cases in Neale and SAIGE for CAD, schizophrenia, and T2D. We note that although the eQTLGen whole blood sample size is much larger than the GTEx whole blood sample size, the identified cis-eQTLs are based on a meta-analysis of 37 different cohorts and therefore heterogenous cell type-composition effects might persist. Finally, some tissues in the GTEx project involve an in vitro manipulation such as EBV-transformed lymphocytes. We identified co-clusters and significant pathways within EBV-transformed lymphocytes that warrant cautious interpretation.

In this study, the intention was to report colocalization signals and identify clusters of phenotypes sharing colocalized genes at the tissue level. One possible research avenue could exploit the sharing of eQTLs among biologically related tissues to improve statistical power to detect colocalized genes at the tissue level. The recently published method JTI^36^ leverages this abundance of shared eQTLs to improve prediction of gene expression levels. This method relies on prediction models of gene expression, and has to be distinguished from colocalization methods. This approach of combining predicted expression data with a colocalization method has been recently proposed^37^.

In conclusion, we showed that the phenome-wide map can be a useful resource to understand gene, phenotype, and tissue links across a wide spectrum of biological classes and diseases. We expect that further interrogation of the phenome-wide map will yield more biological and clinical discoveries.

## Methods

### Datasets

This study uses two resources: (a) the UK Biobank (UKBB) project; and (b) the Genotype-Tissue Expression (GTEx) project. The UKBB project is a prospective EHR-linked cohort with deep genetic and rich phenotypic data collected on ~500,000 middle-aged individuals (aged between 40 and 69 years old) recruited from across the United Kingdom^1^. Ethics approval for the UK Biobank project was obtained from the North West Centre for Research Ethics Committee (11/NW/0382) and all participants provided written informed consent. The GTEx project is a resource database and associated multi-tissue bank aimed at studying the relationship between genetic variation and gene expression in different human tissues^5–7^.

### Colocalization method

We integrated multiple association datasets to assess whether two association signals, one from a genome-wide association study (GWAS) on a phenotype, and the other from expression quantitative trait locus (eQTL) analysis in a tissue, overlap in such a matter that they are consistent with a shared causal gene. This approach, referred to as colocalization, was conducted using coloc2^11^, an enhancement of the previously published method coloc^38^. The coloc method is a Bayesian approach which computes the posterior probability that a genetic variant is both associated with the phenotype and the gene expression level in the tissue. Our coloc2 implementation improves over the original coloc method by (1) aligning eQTL and GWAS summary statistics in each eQTL *cis*-region; (2) estimating the likelihood of mixture proportions of five hypotheses (H_0_: no association, H_1_: GWAS associated only, H_2_: eQTL associated only, H_3_: both associated but not colocalized, H_4_: both associated and colocalized) from genome-wide data. These proportions serve as priors in the empirical Bayesian calculation of the posterior probability of colocalization at each locus. Asymptotic Bayes factors are averaged across three different values of the prior variance term (0.01, 0.1, and 0.5)^39^. We defined a colocalized signal using a posterior probability for H_4_ (PPH4) ≥ 0.80, as described previously^38^.

### GWAS and eQTL summary statistics

coloc2 requires both GWAS summary data and eQTL association summary data. For GWAS data, we used two sets from the UKBB project. The first set of results are GWAS association test statistics publicly available from the Neale lab (Round 1 in 2419 phenotypes). We selected variants with minor allele frequency (MAF) > 0.1%. More details on the data quality control and the full list of phenotypes can be found at www.nealelab.is/uk-biobank. We further used a second set of UKBB GWAS association statistics computed by the SAIGE testing method^40^. In total, 1403 case–control phenotypes (PheCodes) were available. We selected variants with MAF > 0.1%. Full datasets and list of PheCodes can be downloaded at https://www.leelabsg.org/resources. For eQTL association signals, we used data from Analysis V7 of the GTEx project available at https://www.gtexportal.org/home/datasets. We restricted our study to the list of 48 tissues (from 620 donors) having a sample size of at least 80. Cis-eQTLs with MAF > 1% were considered as input for coloc2 (detailed in https://storage.googleapis.com/gtex-public-data/Portal_Analysis_Methods_v7_09052017.pdf available on the GTEx Portal).

Before running coloc2 on the Neale GWAS data, we performed stringent quality control. First, we removed results from phenotypes related to cause of death (since these phenotypes generally had very low number of cases), and we also removed case–control phenotypes with less than 1250 cases (or controls), except phenotypes showing prior gene/locus association as reported in the NHGRI-EBI GWAS catalog (https://www.ebi.ac.uk/gwas/)^41^. The rationale for excluding phenotypes with less than 1250 cases (or controls) is based on the recommendation by Neale to keep only variants with at least 25 minor alleles in the sample of cases (or controls), in order to avoid inflation in association test statistics due to extreme case–control ratio imbalance and ensuring reliable *P* value computation as detailed in http://www.nealelab.is/blog/2017/9/11/details-and-considerations-of-the-uk-biobank-gwas. With the Neale GWAS data, we retained coloc2 results for 496 continuous and binary phenotypes. In the same vein, before running coloc2 with SAIGE association data, we excluded case–control phenotypes with less than 200 cases, as recommended by the authors^40^. With SAIGE data, we generated coloc2 results for 915 case–control (PheCode) phenotypes.

### Construction and descriptive statistics of bipartite networks

To construct the phenome-wide map of genes and phenotypes using the colocalized data of 9151 genes and 1411 phenotypes, we created a bipartite network for each tissue. In brief, a bipartite network, also called a two-mode network, is a network in which nodes of one mode (i.e., type) are only connected to nodes of the other mode (for a review, see refs. ^8,9^). Associations between phenotypes and genes are indicated by links or edges between them.

To characterize and compare colocalization results across tissues, we computed descriptive statistics adapted to bipartite networks. We computed the average degree of colocalized genes and phenotypes in each tissue. The degree of a given gene (respectively, phenotype) is simply the number of unique phenotypes (respectively, genes) connected to it in the network, the average being taken over the total number of genes (respectively, phenotypes)^12^.

### biLouvain clustering algorithm

For each tissue, it is expected that the bipartite network of coloc2 results will tend to cluster in small groups of related phenotypes with their causally associated genes. To uncover clustering within each network, we applied the biLouvain clustering algorithm, an extension of the unipartite Louvain clustering algorithm. This algorithm identifies co-clusters, also called communities, of non-overlapping genes and phenotypes through maximization of a modularity score adapted to bipartite networks (see ref. ^10^ for details). To decrease the overall computational time of the algorithm, we opted for the Fuse preprocessing step before running the co-clustering step per se. In rare occasions, this fusing step incurred some loss in clustering quality, hence resulting in missing edges in the co-cluster (e.g., Supplementary Fig. 5).

### Comparison between eQTLGen and GTEX

The eQTLGen consortium identified cis-eQTLs with MAF > 1% based on a meta-analysis of 37 different cohorts in up to 31,684 individuals^23^. Every SNP-gene combination within a distance <1 Mb from the center of the gene and tested in at least two cohorts were included. We ran coloc2 using as input the same GWAS summary statistics (Neale and SAIGE) and cis-eQTL variants from the eQTLGen consortium. An overlapping locus between GTEX and eQTLGen was reported if one signal found in one set of colocalization results overlapped within 2 Mb in the other set of results (as a consequence, many genes may appear in the same locus). The biLouvain algorithm was run in eQTLGen colocalization results using the same options as in GTEX results.

### Colocalization with disease case sample size larger than UKBB

In order to assess the effect of using GWAS summary statistics with case sample size in various diseases larger than in UKBB, we downloaded three GWAS meta-analysis case–control datasets as generated by three different consortia: coronary artery disease from CARDIoGRAMplusC4D (60,801 cases and 123,504 controls)^24^, schizophrenia from the Psychiatric Genomics Consortium (36,989 cases and 113,075 controls)^25^, and type 2 diabetes from DIAGRAM (55,005 cases and 400,308 controls, after excluding UKBB participants)^26^. We ran coloc2 by combining each of these three GWAS datasets with the same GTEX cis-eQTL dataset used before (all 48 tissues).

### PANTHER enrichment analysis

To test if biLouvain co-clusters were enriched with some functional gene classes, we selected 183 co-clusters consisting of 10 genes or more, and input them into the online PANTHER enrichment analysis tools^13,14^. We applied Fisher overrepresentation tests on four different annotation types: Biological process (2064 Gene Ontology (GO) terms), Cellular component (520 GO terms), Molecular function (532 GO terms), and 164 different Pathways. For each co-cluster and each annotation type, we took the minimal *P* value of all Fisher tests, and plotted it against the expected minimal *P* value under the null hypothesis of no enrichment. We assumed that *P* values within each annotation type are independently distributed uniformly over the interval (0,1), which represents a conservative approach. Note that the minimal *P* value of *n* independent *P* values from a Uniform (0,1) is not uniformly distributed under the null: its cumulative density function is instead given by

1 $${{{{{\rm{Prob}}}}}}\left(X\le x\right)=1-{\left(1-x\right)}^{n},\,0 \, < \, x \, < \, 1.$$

In each panel of Fig. 3, we plotted a straight line with slope equal to 1, which crosses the *y* axis at *x* = (observed 1st quartile – expected 1st quartile) using the above expected cumulative density function. Gene enrichment was deemed significant if the minimal *P* value was less than 0.05/(183 × 4) = 6.8 × 10^−5^.

### Reporting summary

Further information on research design is available in the Nature Research Reporting Summary linked to this article.

## Supplementary information

Supplementary Information

Description of Additional Supplementary Files

Supplementary Data 1

Supplementary Data 2

Supplementary Data 3

Supplementary Data 4

Supplementary Data 5

Supplementary Data 6

Supplementary Data 7

Supplementary Data 8

Supplementary Data 9

Supplementary Data 10

Reporting Summary

## Data Availability

All colocalized results analyzed in this study are available through a R Shiny app called biPheMap at https://rstudio-connect.hpc.mssm.edu/biPheMap/. This research has been conducted using the UK Biobank Resource under Application Number “16218”. UK Biobank data is available to researchers upon approval of an application form at https://www.ukbiobank.ac.uk/. The GTEx Analysis V7 dataset can be freely downloaded at https://www.gtexportal.org/home/datasets. At the time of our colocalization analysis, we utilized Round 1 of Benjamin Neale’s lab GWAS summary statistics in the UK Biobank. The Round 1 results are no longer accessible and has since been replaced by a more recent Round 2, which can be freely downloaded at http://www.nealelab.is/uk-biobank. Seunggeun Lee’s lab GWAS summary statistics in the UK Biobank using SAIGE can be freely downloaded at https://www.leelabsg.org/resources. The full cis-eQTL summary statistics from the eQTLGen Consortium are publicly available at https://www.eqtlgen.org/cis-eqtls.html. GWAS summary statistics from CARDIoGRAMplusC4D (http://www.cardiogramplusc4d.org/data-downloads/), the Psychiatric Genomics Consortium (https://pgc.unc.edu/for-researchers/download-results/), and DIAGRAM (https://diagram-consortium.org/downloads.html) are publicly available and can be freely downloaded.

## Code availability

R codes to run coloc2 are available at https://github.com/Stahl-Lab-MSSM. The biLouvain algorithm may be downloaded and installed by following instructions at https://github.com/paolapesantez/biLouvain. The PANTHER classification system is available at http://www.pantherdb.org/. R software and packages (https://cran.r-project.org/) were used to analyze the data and generate the figures, except Fig. 1, which contains graphical elements from a free version of Canva (https://www.canva.com/en/).
