# Supplementary material for: A tissue-level phenome-wide network map of colocalized genes and phenotypes in the UK Biobank
Source: Commun Biol. 2022 Aug 20;5:849. doi: 10.1038/s42003-022-03820-z (PMC9392744; doi:10.1038/s42003-022-03820-z)
Supplement: Supplementary file 1 — Supplementary Information [file 42003_2022_3820_MOESM1_ESM.pdf]

Supplementary Figure 1.

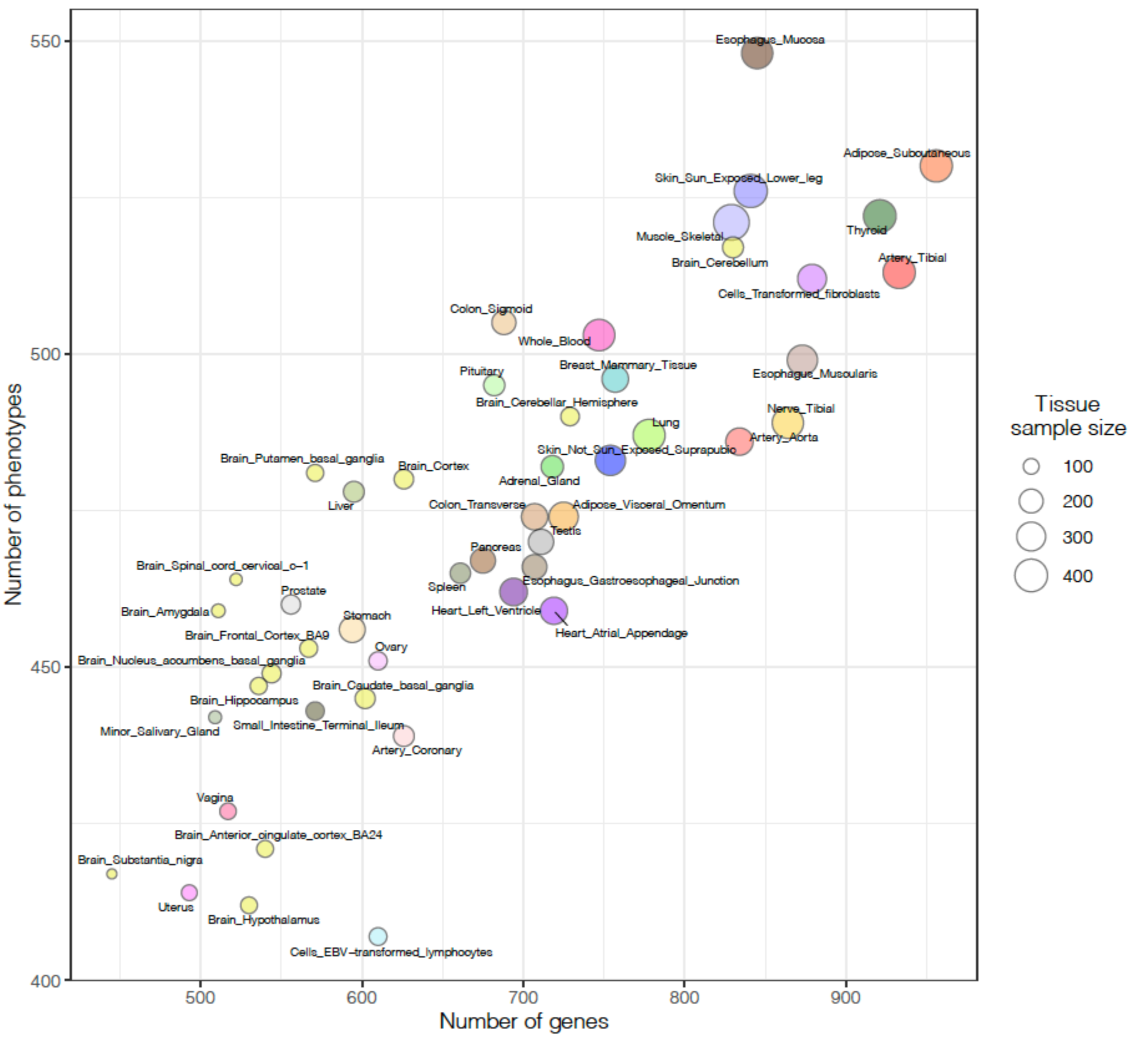

Bubble plot of the number of colocalization signals for each tissue.
Number of genes and number of phenotypes are given for each tissue, and the size of the bubble is proportional to the tissue sample size in the GTEx project.

Supplementary Figure 2.

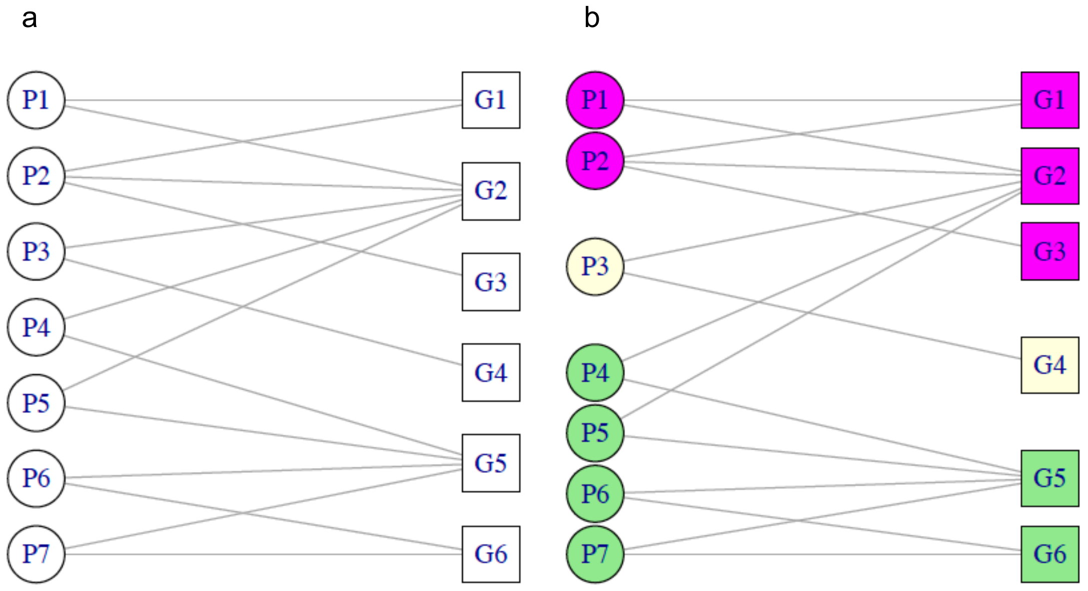

Graphical representation of a bipartite graph.
a) Bipartite graph with 7 phenotypes (P1 to P7) and 6 genes (G1 to G6). **b**) Algorithm biLouvain identifies 3 co-clusters made of phenotypes and genes: {P1,P2,G1,G2,G3} in magenta, {P3,G4} in light yellow, and {P4,P5,P6,P7,G5,G6} in light green.

Supplementary Figure 3.

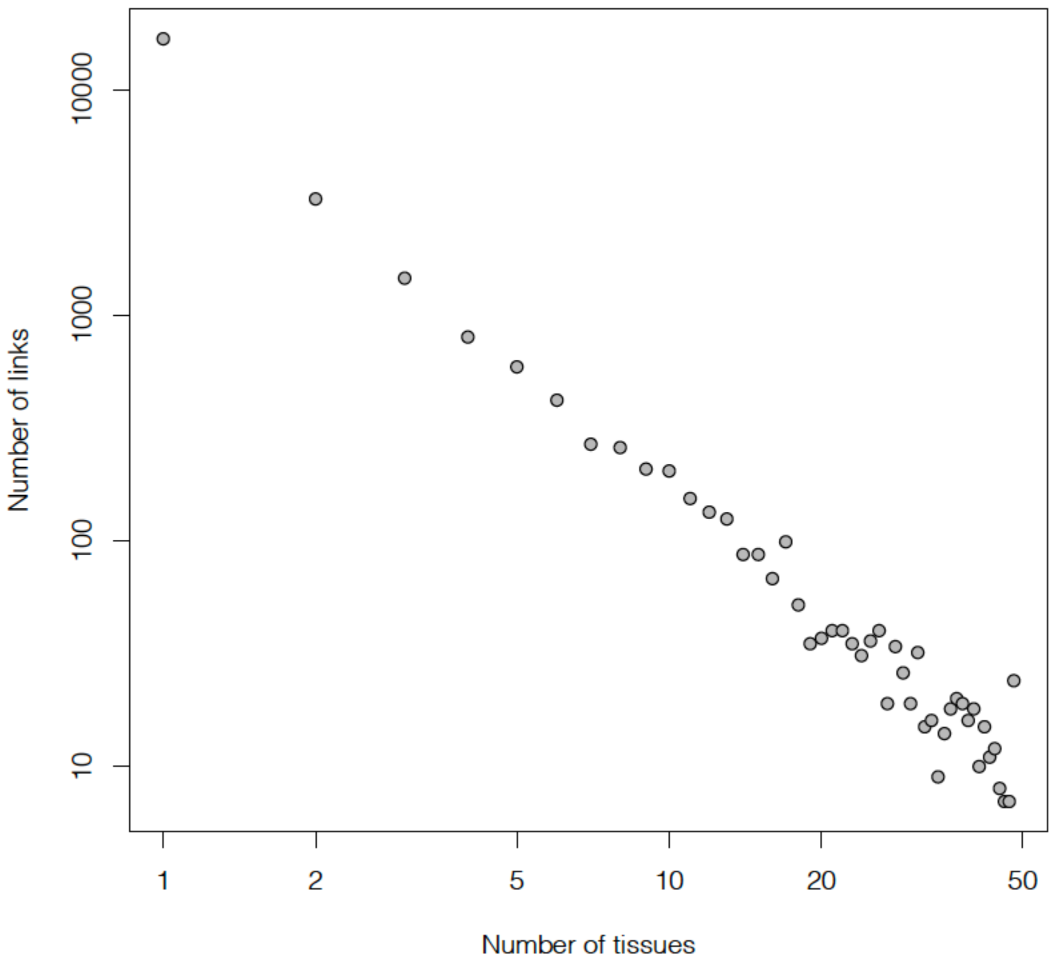

Number of gene-phenotype links observed in number of tissues.
Distribution of the number of times a link (phenotype-gene pair) appears across all tissues (varies from 1 to 48). Both axes are displayed in log scale.

Supplementary Figure 4.

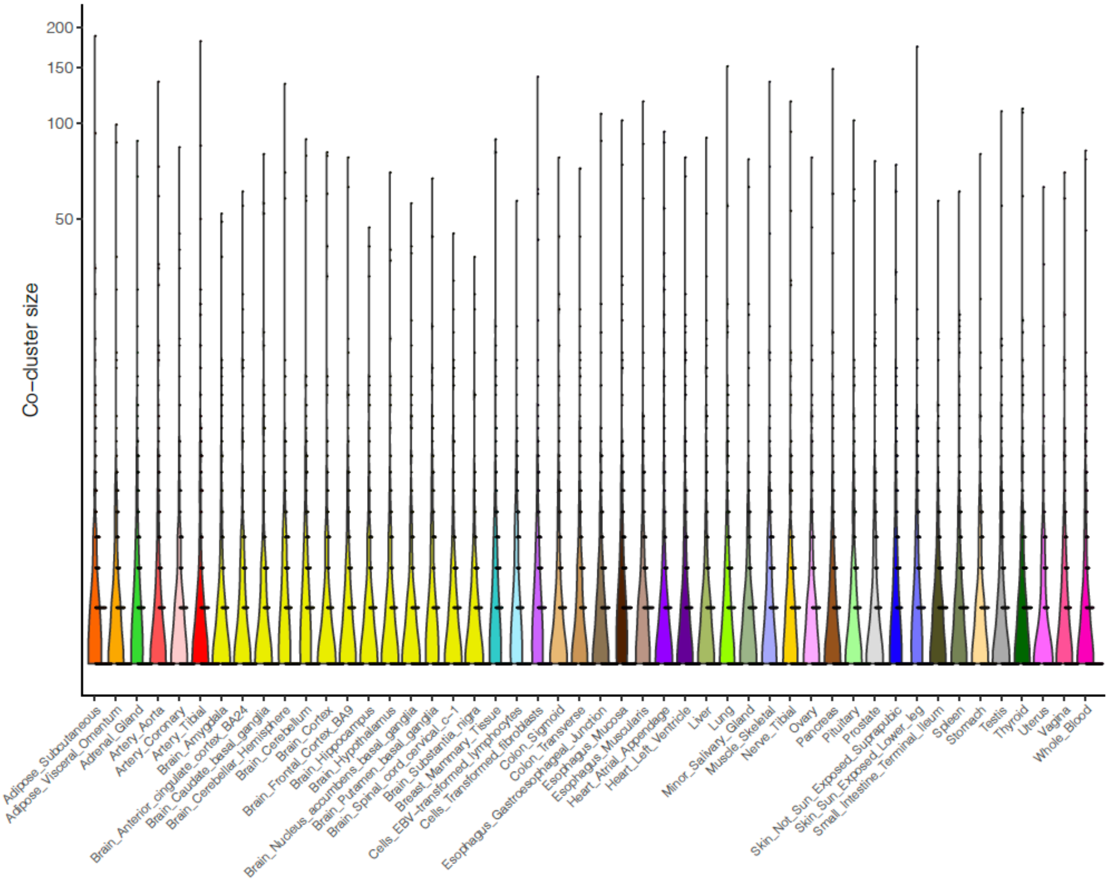

Violin plot of co-cluster size (number of phenotypes + number of genes) for each tissue.

Individual data points are overlaid (off center right) on each violin plot to make them more visible. Many points are stacked onto each other for smaller cluster size. Y-axis is displayed in log scale.

Supplementary Figure 5.

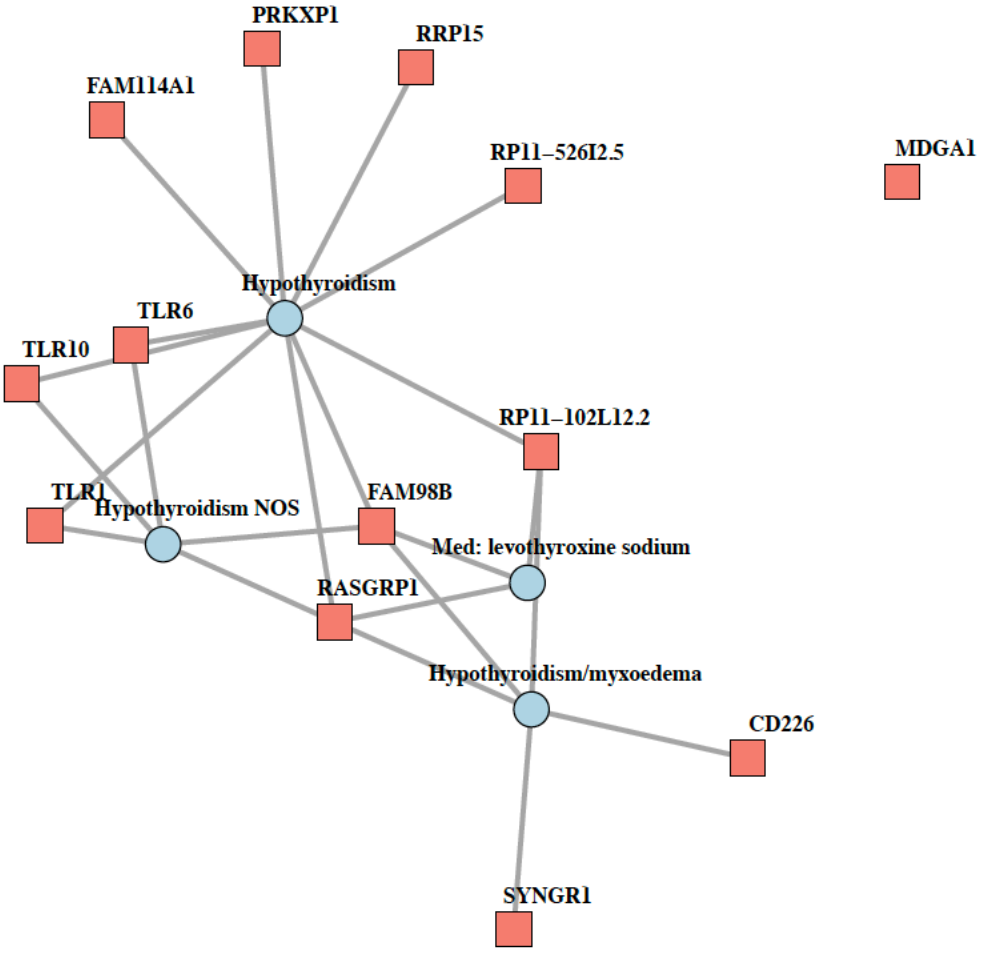

Plot of subgraph induced by co-cluster 111 in tissue Cells - EBV-transformed lymphocytes.
Genes and phenotypes are represented by salmon squares and light blue circles, respectively. Med: Medication; NOS: Not otherwise specified.

Supplementary Figure 6.

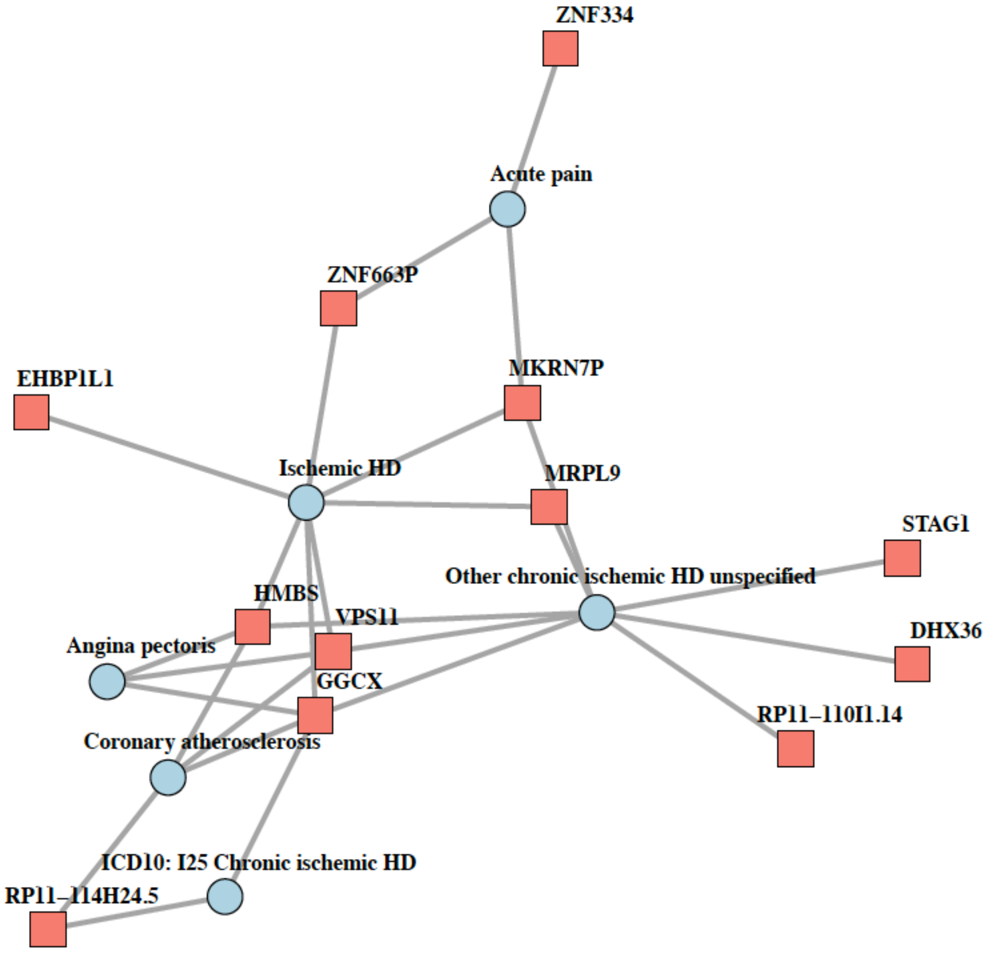

Plot of subgraph induced by co-cluster 161 in tissue Heart - Left Ventricle. Genes and phenotypes are represented by salmon squares and light blue circles, respectively. HD: Heart Disease.

Supplementary Figure 7.

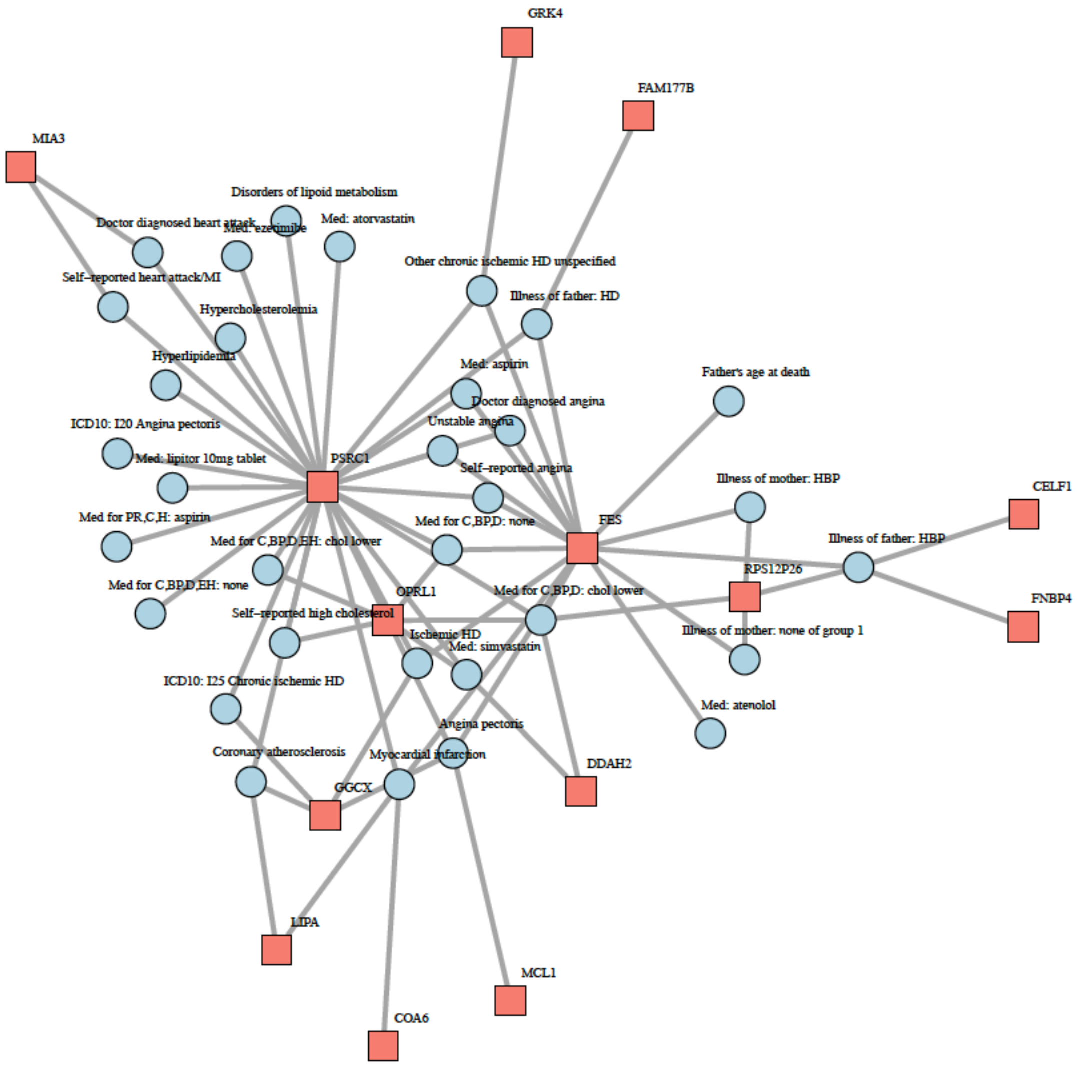

Plot of subgraph induced by co-cluster 11 in tissue Whole blood.
Genes and phenotypes are represented by salmon squares and light blue circles, respectively. Illness of mother group 1 refers to UKBB data-field 20110 which comprises heart disease, stroke, high blood pressure, chronic bronchitis/emphysema, Alzheimer's disease/dementia, diabetes; Med: medication; Med for C,BP,D: Medication for cholesterol, blood pressure, diabetes; HD: heart disease; Med for C,BP,D, EH: Medication for cholesterol, blood pressure, diabetes, or take exogenous hormones; Med for PR,C,H: Medication for pain relief, constipation, heartburn; MI: myocardial infarction; HBP: high blood pressure; chol lower: cholesterol lowering.
