# Supplementary material for: A tissue-level phenome-wide network map of colocalized genes and phenotypes in the UK Biobank
Source: Commun Biol. 2022 Aug 20;5:849. doi: 10.1038/s42003-022-03820-z (PMC9392744; doi:10.1038/s42003-022-03820-z)
Supplement: Supplementary file 2 — Description of Additional Supplementary Files [file 42003_2022_3820_MOESM2_ESM.pdf]

Description of Additional Supplementary Files

File name: Supplementary Data 1.

Description: Colocalization results across the 48 selected tissues in GTEx (9,151 unique colocalized genes for 1,411 unique phenotypes).

Each row represents a colocalized signal between the gene and the phenotype in the corresponding tissue. In the Phenotype column, the prefix “N_” stands for Neale’s GWAS association statistics, while “S_” stands for SAIGE GWAS association statistics. Number of SNPs used by coloc2, along with PPH4 values, are displayed for each colocalized signal.

File name: Supplementary Data 2.

Description: Descriptive statistics of tissue-level bipartite networks.

For each bipartite network, the number of phenotypes, genes and links are reported, along with the average degree across all phenotypes and all genes, respectively.

File name: Supplementary Data 3.

Description: Colocalization results aggregated over tissues.

Each row represents a unique colocalized signal between the gene and the phenotype, and reports in how many tissues this signal is found, along with the list of these tissues. In the Phenotype column, the prefix “N_” stands for Neale’s GWAS association statistics, while “S_” stands for SAIGE GWAS association statistics.

File name: Supplementary Data 4.

Description: List of all co-clusters found by the biLouvain algorithm for each tissue-level bipartite network.

The number of phenotypes, the number of genes and the co-cluster size in each co-cluster are provided in the last three columns (co-cluster size = number of phenotypes + number of genes). In the Phenotype column, the prefix “N_” stands for Neale’s GWAS association statistics, while “S_” stands for SAIGE GWAS association statistics.

File name: Supplementary Data 5.

Description: Colocalization results in eQTLGen.

Each row represents a colocalized signal between the gene and the phenotype. In the Phenotype column, the prefix “N_” stands for Neale’s GWAS association statistics, while “S_” stands for SAIGE GWAS association statistics. Number of SNPs used by coloc2, along with PPH4 values, are displayed for each colocalized signal.

File name: Supplementary Data 6.

Description: Overlapping loci between GTEX and eQTLGen colocalization results.

Each row displays a phenotype for which a GTEX colocalized signal overlaps with an eQTLGen colocalized signal. Overlapping signals might contain more than one gene. In the Phenotype column, the prefix “N_” stands for Neale’s GWAS association statistics, while “S_” stands for SAIGE GWAS association statistics.

File name: Supplementary Data 7.

Description: List of all co-clusters found by the biLouvain algorithm in eQTLGen.

The number of phenotypes and genes in each co-cluster are provided in the last two columns (number of phenotypes and number of genes). In the Phenotype column, the prefix “N_” stands for Neale’s GWAS association statistics, while “S_” stands for SAIGE GWAS association statistics.

File name: Supplementary Data 8.

Description: Colocalization results across the 48 GTEX tissues for coronary artery disease (CAD) from the CARDIoGRAMplusC4D consortium.

Each row represents a colocalized signal between the gene and CAD in the corresponding tissue. Number of SNPs used by coloc2, along with PPH4 values, are displayed for each colocalized signal.

File name: Supplementary Data 9.

Description: Colocalization results across the 48 GTEX tissues for schizophrenia from the Psychiatric Genomics Consortium.

Each row represents a colocalized signal between the gene and schizophrenia in the corresponding tissue. Number of SNPs used by coloc2, along with PPH4 values, are displayed for each colocalized signal.

File name: Supplementary Data 10.

Description: Colocalization results across the 48 GTEX tissues for type 2 diabetes (T2D) from the DIAGRAM consortium.

Each row represents a colocalized signal between the gene and T2D in the corresponding tissue. Number of SNPs used by coloc2, along with PPH4 values, are displayed for each colocalized signal.
