# Supplementary material for: A tissue-level phenome-wide network map of colocalized genes and phenotypes in the UK Biobank
Source: Commun Biol. 2022 Aug 20;5:849. doi: 10.1038/s42003-022-03820-z (PMC9392744; doi:10.1038/s42003-022-03820-z)
Supplement: Supplementary file 13 — Reporting Summary [file 42003_2022_3820_MOESM13_ESM.pdf]

nature portfolio

Corresponding author(s): Ron Do

Last updated by author(s): Jul 19, 2022

Reporting Summary

Nature Portfolio wishes to improve the reproducibility of the work that we publish. This form provides structure for consistency and transparency in reporting. For further information on Nature Portfolio policies, see our Editorial Policies and the Editorial Policy Checklist.

Statistics

For all statistical analyses, confirm that the following items are present in the figure legend, table legend, main text, or Methods section.

n/a	Confirmed	
☐	☒	The exact sample size (*n*) for each experimental group/condition, given as a discrete number and unit of measurement
☒	☐	A statement on whether measurements were taken from distinct samples or whether the same sample was measured repeatedly
☐	☒	The statistical test(s) used AND whether they are one- or two-sided<br>*Only common tests should be described solely by name; describe more complex techniques in the Methods section.*
☐	☒	A description of all covariates tested
☐	☒	A description of any assumptions or corrections, such as tests of normality and adjustment for multiple comparisons
☐	☒	A full description of the statistical parameters including central tendency (e.g. means) or other basic estimates (e.g. regression coefficient) AND variation (e.g. standard deviation) or associated estimates of uncertainty (e.g. confidence intervals)
☐	☒	For null hypothesis testing, the test statistic (e.g. *F*, *t*, *r*) with confidence intervals, effect sizes, degrees of freedom and *P* value noted<br>*Give P values as exact values whenever suitable.*
☒	☐	For Bayesian analysis, information on the choice of priors and Markov chain Monte Carlo settings
☒	☐	For hierarchical and complex designs, identification of the appropriate level for tests and full reporting of outcomes
☐	☒	Estimates of effect sizes (e.g. Cohen's *d*, Pearson's *r*), indicating how they were calculated

Our web collection on statistics for biologists contains articles on many of the points above.

Software and code

Policy information about availability of computer code

Data collection	No software was used to collect data. We downloaded publicly available datasets on the Internet.
Data analysis	R codes to run coloc2 are available at https://github.com/Stahl-Lab-MSSM. The biLouvain algorithm may be downloaded and installed by following instructions at https://github.com/paolapesantez/biLouvain. The PANTHER classification system is available at http://www.pantherdb.org/. R software and packages (https://cran.r-project.org/) were used to analyze the data and generate the figures, except Figure 1 which contains graphical elements from a free version of Canva (https://www.canva.com/en/).

For manuscripts utilizing custom algorithms or software that are central to the research but not yet described in published literature, software must be made available to editors and reviewers. We strongly encourage code deposition in a community repository (e.g. GitHub). See the Nature Portfolio guidelines for submitting code & software for further information.

Data

Policy information about availability of data

All manuscripts must include a data availability statement. This statement should provide the following information, where applicable:

- Accession codes, unique identifiers, or web links for publicly available datasets
- A description of any restrictions on data availability
- For clinical datasets or third party data, please ensure that the statement adheres to our policy

All colocalized results analyzed in this study are available through a R Shiny app called biPheMap at https://rstudio-connect.hpc.mssm.edu/biPheMap/. This research

March 2021

has been conducted using the UK Biobank Resource under Application Number '16218'. UK Biobank data is available to researchers upon approval of an application form at https://www.ukbiobank.ac.uk/. The GTEx Analysis V7 dataset can be freely downloaded at https://www.gtexportal.org/home/datasets. At the time of our colocalization analysis, we utilized Round 1 of Benjamin Neale's lab GWAS summary statistics in the UK Biobank. The Round 1 results are no longer accessible and has since been replaced by a more recent Round 2 which can be freely downloaded at http://www.nealelab.is/uk-biobank. Seunggeun Lee's lab GWAS summary statistics in the UK Biobank using SAIGE can be freely downloaded at https://www.leelabsg.org/resources. The full cis-eQTL summary statistics from the eQTLGen Consortium are publicly available at https://www.eqtlgen.org/cis-eqtls.html. GWAS summary statistics from CARDIoGRAMplusC4D (http://www.cardiogramplusc4d.org/data-downloads/), the Psychiatric Genomics Consortium (https://pgc.unc.edu/for-researchers/download-results/), and DIAGRAM (https://diagram-consortium.org/downloads.html) are publicly available and can be freely downloaded.

Human research participants

Policy information about studies involving human research participants and Sex and Gender in Research.

Reporting on sex and gender	Some tissues in GTEx have been collected in only one sex, e.g. prostate in males and ovary in females. Also, some phenotypes collected in UK Biobank are sex-specific, e.g. "self-reported prostate cancer" (Field ID: 20001-1044) or "age when periods started (menarche)" (Field ID: 2714). As a result, some colocalized signals found in our study are relevant to only one sex.
Population characteristics	Our biPheMap rely on the UK Biobank project and the GTEx V7 cis-eQTL dataset. UK Biobank is a prospective EHR-linked cohort with deep genetic and rich phenotypic data collected on approximately 500,000 middle-aged individuals (aged between 40 and 69 years old) recruited from across the United Kingdom (Bycroft et al. Nature 2018). As for the GTEx project, Lonsdale et al. (Nature Genetics 2013) mention that "donors of either sex from any ancestry group are eligible if they are aged 21–70 and if biospecimen collection can start within 24 h of death."
Recruitment	Details of UK Biobank recruitment can be found in DOI: 10.1038/s41586-018-0579-z. Details of the GTEx tissue design and collection is provided in DOI: 10.1038/ng.2653. eQTLGen, CARDIoGRAMplusC4D, Psychiatric Genomics Consortium and DIAGRAM are all consortia, and recruitment of every cohort included in each consortium can be found in DOI: 10.1038/s41588-021-00913-z, DOI: 10.1038/ng.3396, DOI: 10.1038/nature13595 and DOI: 10.1038/s41588-018-0241-6, respectively.
Ethics oversight	Ethics approval are reported in the following publications:<br>UK Biobank: DOI: 10.1038/s41586-018-0579-z<br>GTEX: DOI: 10.1038/ng.2653<br>eQTLGen: DOI: 10.1038/s41588-021-00913-z<br>CARDIoGRAMplusC4D: DOI: 10.1038/ng.3396<br>Psychiatric Genomics Consortium: DOI: 10.1038/nature13595<br>DIAGRAM: DOI: 10.1038/s41588-018-0241-6

Note that full information on the approval of the study protocol must also be provided in the manuscript.

Field-specific reporting

Please select the one below that is the best fit for your research. If you are not sure, read the appropriate sections before making your selection.

☒ Life sciences ☐ Behavioural & social sciences ☐ Ecological, evolutionary & environmental sciences

For a reference copy of the document with all sections, see nature.com/documents/nr-reporting-summary-flat.pdf

Life sciences study design

All studies must disclose on these points even when the disclosure is negative.

Sample size	No sample size calculation was done prior to data analysis. Our colocalization results rely on genome-wide association studies (GWAS) in the UK Biobank and expression quantitative trait loci (eQTL) from the GTEx project. To ensure sufficient statistical power to identify true colocalization signals, we selected variants with minor allele frequency (MAF) > 0.1% from Neale lab and Lee lab (SAIGE) GWAS summary statistics. We restricted our study to the list of 48 tissues having a sample size of at least 80 in the GTEx project version V7, and cis-eQTLs were retained if MAF > 1%. To minimize false positive colocalization signals, we kept only signals with PPH4 >= 0.80. Details can be found in the Methods section.
Data exclusions	We removed phenotypes with less than 1,250 cases (or controls), except when these phenotypes showed prior gene/locus association in the NHGRI-EBI GWAS catalog. We followed a recommendation made by Neale to avoid over-inflation in association p-values. We applied the same rationale to PheCodes from Lee lab by excluding case-control phenotypes with less than 200 cases. More details can be found in the Methods section.
Replication	We compared the colocalized loci found in our biPheMap using a different cis-eQTL dataset and three large case-control GWAS datasets. First, we ran coloc2 combining the eQTLGen consortium dataset (cis-eQTLs with MAF > 1% in up to 31,684 individuals) with Neale and SAIGE datasets. Then, we ran coloc2 combining the GTEx V7 dataset with GWAS summary statistics for coronary artery disease (CARDIoGRAMplusC4D: 60,801 cases and 123,504 controls), schizophrenia (Psychiatric Genomics Consortium: 36,989 cases and 113,075 controls), and type 2 diabetes (DIAGRAM: 55,005 cases and 400,308 controls). More details can be found in the Methods section.
Randomization	This is not relevant to our study since GWAS signals and eQTLs have been identified in large biobanks which are observational by nature.

Blinding	No blinding was deemed necessary since we did not have any control in the recruitment process of either the UK Biobank or the GTEx project.

Reporting for specific materials, systems and methods

We require information from authors about some types of materials, experimental systems and methods used in many studies. Here, indicate whether each material, system or method listed is relevant to your study. If you are not sure if a list item applies to your research, read the appropriate section before selecting a response.

Materials & experimental systems

n/a	Involved in the study	
☒	☐	Antibodies
☒	☐	Eukaryotic cell lines
☒	☐	Palaeontology and archaeology
☒	☐	Animals and other organisms
☒	☐	Clinical data
☒	☐	Dual use research of concern

Methods

n/a	Involved in the study	
☒	☐	ChIP-seq
☒	☐	Flow cytometry
☒	☐	MRI-based neuroimaging
